# Structural biology of shelterin and telomeric chromatin: the pieces and an unfinished puzzle

**DOI:** 10.1042/BST20230300

**Published:** 2024-08-07

**Authors:** Hongmiao Hu, Helen Linwen Yan, Thi Hoang Duong Nguyen

**Affiliations:** MRC Laboratory of Molecular Biology, Cambridge, U.K.

**Keywords:** shelterin, telomeres, telomeric chromatin

## Abstract

The six-subunit shelterin complex binds to mammalian telomeres and protects them from triggering multiple DNA damage response pathways. The loss of this protective function by shelterin can have detrimental effects on cells. In this review, we first discuss structural studies of shelterin, detailing the contributions of each subunit and inter-subunit interactions in protecting chromosome ends. We then examine the influence of telomeric chromatin dynamics on the function of shelterin at telomeres. These studies provide valuable insights and underscore the challenges that future research must tackle to attain high-resolution structures of shelterin.

## Introduction

In most eukaryotes, telomeric DNA comprises tandem double-stranded (ds) G-rich telomeric DNA repeats (GGTTAG in mammals) and a 3′ single-stranded (ss) G-rich overhang. This structure resembles DNA breaks and necessitates mechanisms to differentiate telomeres from breaks to maintain genome stability [1]. Mammalian cells employ shelterin, a six-membered protein complex, to bind to telomeric DNA and protect chromosome ends from the activation of DNA damage response (DDR) pathways [2,3]. Besides providing end-protection, shelterin also regulates telomerase, a specialised reverse transcriptase that extends telomeric repeats to counteract telomere shortening caused by incomplete genome replication [4].

**Figure 1.**
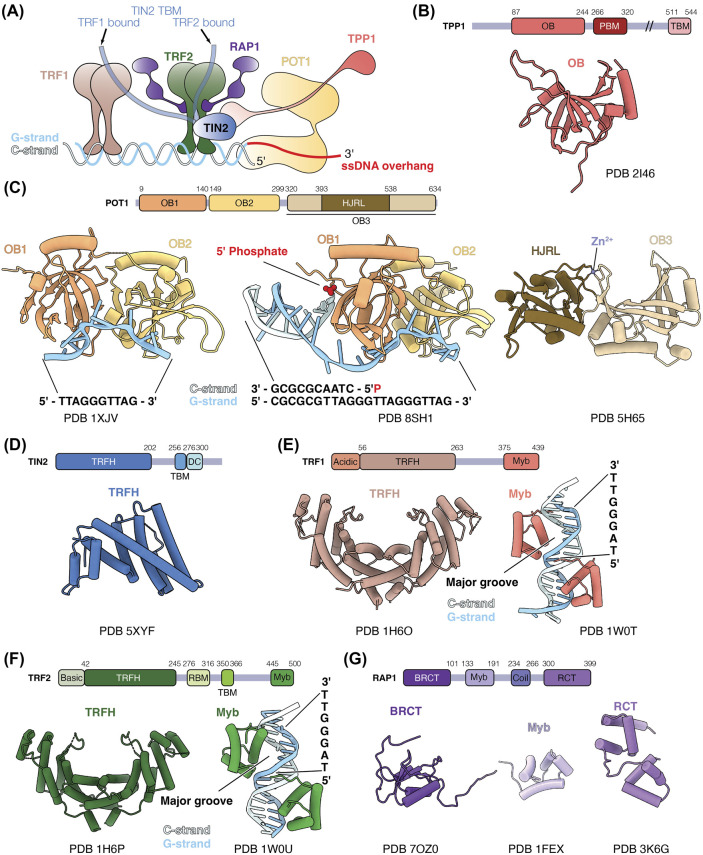
Shelterin and its subunits. (**A**) Schematic of shelterin and its interactions with telomeric DNA. Subunits are not drawn to scale; and subunit stoichiometry is hypothetical. (**B**) Domain architecture of TPP1 (top) and structure of the OB domain of TPP1 (bottom) (PDB 2I46) [16]. OB, oligosaccharide/oligonucleotide binding; PBM, POT1-binding motif; and TBM, TIN2-binding motif. (**C**) Domain architecture of POT1 (top) and structures of POT1 domains (bottom) (PDB 1XJV, 8SH1 and 5H65) [8,47,48]. OB, oligosaccharide/oligonucleotide binding; and HJRL, Holliday junction resolvase-like. (**D**) Domain architecture of TIN2 (top) and structure of the TRFH domain of TIN2 (bottom) (PDB 5XYF) [52]. TRFH, telomere repeat factor homology; TBM, TRFH-binding motif; and DC, mutations-in-dyskeratosis congenita. (**E**) Domain architecture of TRF1 (top) and structures of the TRFH and Myb domains of TRF1 (bottom) (PDB 1H6O and 1W0T) [24,53]. TRFH, telomere repeat factor homology. (**F**) Domain architecture of TRF2 (top) and structures of the TRFH and Myb domains of TRF2 (bottom) (PDB 1H6P and 1W0U) [24,53]. TRFH, telomere repeat factor homology; RBM, RAP1-binding motif; and TBM, TIN2-binding motif. (**G**) Domain architecture of RAP1 (top) and structures of the BRCT, Myb and RCT domains of RAP1 (bottom) (PDB 7OZ0, 1FEX and 3K6G) [32,70]. BRCT, BRCA1 carboxy terminal; coil, a coiled region; and RCT, RAP1 carboxy terminal. Dash lines represent disordered regions.

Shelterin consists of TRF1, TRF2, RAP1, TIN2, TPP1, and POT1 (Figure 1A). Extensive studies have elucidated the distinct roles of each subunit in telomere maintenance and their interactions with each other and other accessory proteins [2,3]. POT1 binds ss telomeric DNA and the telomeric ds-ss DNA junction, suppressing ATR kinase activation and DDR at telomeres by excluding replication protein A (RPA) [5–8]. TPP1 recruits telomerase to telomeres by direct interactions with the telomerase reverse transcriptase (TERT) subunit [9–15]. POT1 and TPP1 form a heterodimer, which enhances telomerase processivity *in vitro* [16]. By sequestering the 3′ telomeric overhang from telomerase, POT1 also negatively regulates telomerase [17–19]. Partial disruption of POT1-TPP1 interaction results in longer, fragile telomeres, likely due to increased telomerase access at telomeres [20]. How POT1-TPP1 switches between telomerase recruitment and telomerase inhibition remains unclear.

TRF1 and TRF2 bind telomeric dsDNA [21–24]. TRF1 aids telomere replication by preventing replication fork stalling and ATR pathway activation via TIN2-dependent recruitment of TPP1-POT1 [25,26]. In contrast, TRF2 inhibits non-homologous end joining and ATM-dependent DDR pathways [7,27,28]. One proposed mechanism for DDR inhibition by TRF2 is by promoting the formation of telomeric loops (T-loops), in which the 3′ ss overhang inserts into the ds region of telomeres [29–31].

RAP1, an accessory protein to TRF2, is crucial for suppressing homology-directed repair and protecting critically short telomeres from chromosomal fusion [32–36]. TIN2 interacts with TPP1, TRF1, and TRF2, bridging ss and ds DNA binding subunits within shelterin [37–41]. The POT1-TPP1-TIN2 complex enhances telomerase processivity, with the inclusion of TIN2 amplifying this effect compared with POT1-TPP1 alone [14,42].

In higher eukaryotes, telomeric DNA is packaged into nucleosomes, forming high-order chromatin structures [43,44]. Although interactions between shelterin and telomeric chromatin are not fully understood, emerging evidence suggests that telomeric chromatin modulates shelterin action at telomeres and vice versa [45,46].

In this review, we will provide an overview of structural studies of shelterin and its interactions with telomeric chromatin. These studies shed light on the dynamic nature of shelterin and its interactions, which enable shelterin to perform a wide range of tasks at telomeres.

## Structural studies of shelterin subunits

### POT1-TPP1 — the single-stranded telomeric DNA binding unit

POT1 consists of three oligosaccharide/oligonucleotide binding (OB) domains, known as OB1, OB2 and OB3, and a Holliday junction resolvase-like (HJRL) domain nestled within the OB3 domain (Figure 1C). TPP1 comprises an N-terminal (OB) domain, a POT1-binding motif (PBM) and a TIN2-binding motif (TBM) (Figure 1B).

POT1 binds ss telomeric DNA through its OB1 and OB2 domains [47]. The crystal structure of POT1 OB1 and OB2 bound to a ssDNA with a sequence of TTAGGGTTAG shows that OB1 domain binds the TTAGGG motif and OB2 domain binds TTAG (Figure 1C) [47]. The ssDNA adopts an extended conformation, bending across the two OB domains. POT1 affinity for telomeric ssDNA is greater for the sequence terminating in GGTTAG compared with TTAGGG, which aligns with the natural sequence resulting from repeat addition by telomerase [47].

A crystal structure of POT1 bound to a telomeric ds-ss DNA junction was recently reported [8]. In telomeric DNA, the strand complementary to the G-rich strand is termed the C-strand. The structure unveiled intricate electrostatic and stacking interactions between the 5′-phosphate of the terminal cytosine on the C-strand and a cavity in the POT1 OB1 domain termed the ‘POT-hole’ (Figure 1C) [8]. Notably, POT1 exhibits a preference for binding to the ds-ss junction terminating with 5′-TAG-3′/3′-ATC-5′ over the other telomeric permutations. Mutating the POT-hole impairs POT1 binding to telomeres and its ability to suppress DDR at telomeres [8]. This underscores the role of POT1 recognition of the ds-ss telomeric junction in telomere protection.

**Figure 2.**
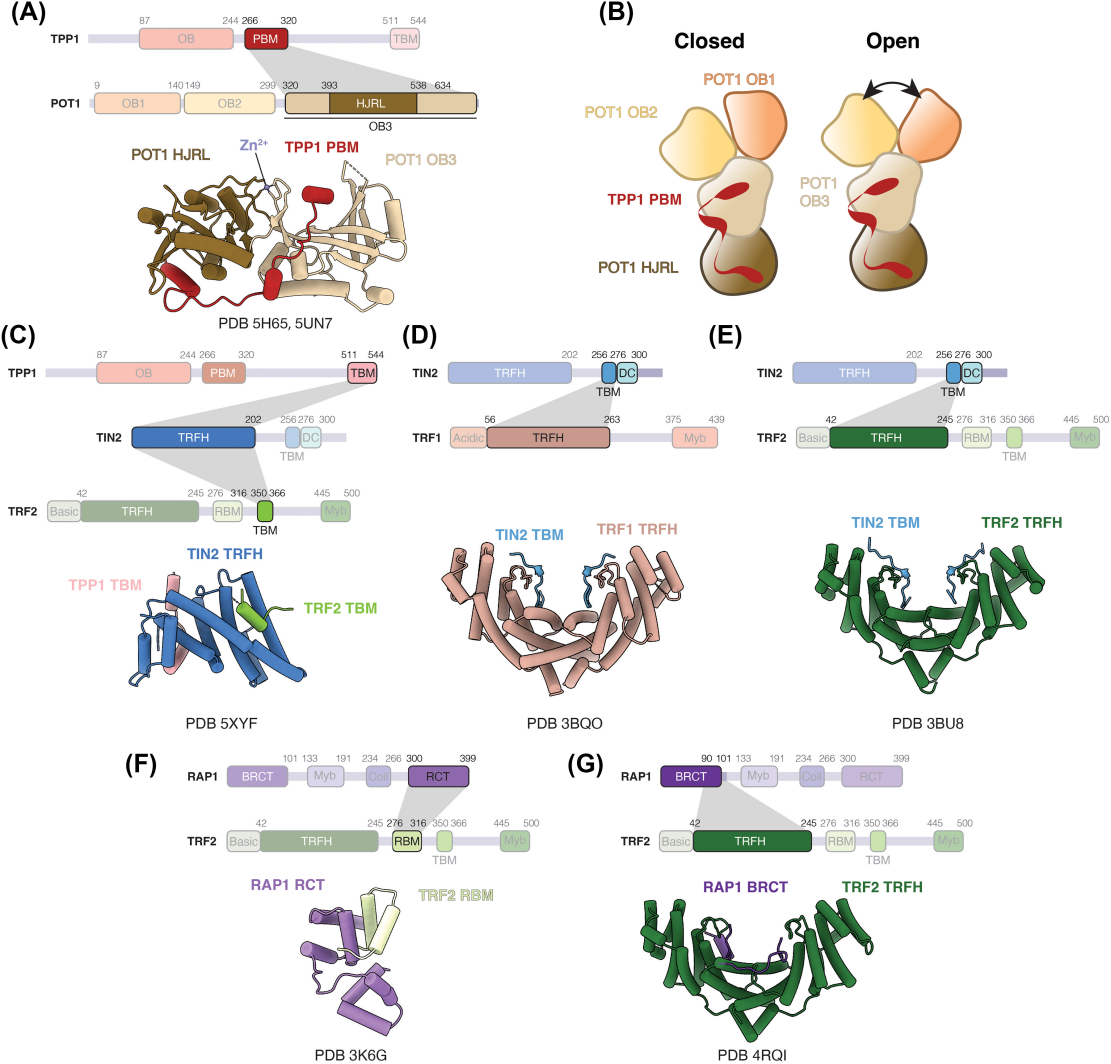
Structural studies of inter-subunit interactions within shelterin. (**A**) Structure of the POT1-binding motif (PBM) of TPP1 bound to the OB3 and HJRL domains of POT1 (PDB 5H65 and 5UN7) [20,48]. (**B**) Schematics depicting the domain organisation of POT1 and TPP1 PBM observed in the cryoEM structure of POT1-TPP1-TIN2 [50]. The OB1 and OB2 domains of POT1 can adopt a closed (left) or open (right) conformation. (**C**) Structure of the telomere repeat factor homology (TRFH) domain of TIN2 bound to the TIN2-binding motif (TBM) of TPP1 and TRF2 (PDB 5XYF) [52]. (**D**) Structure of the TRFH domain of TRF1 bound to the TRFH-binding motif of TIN2 (PDB 3BQO) [51]. (**E**) Structure of the TRFH domain of TRF2 bound to the TRFH-binding motif of TIN2 (PDB 3BU8) [51]. (**F**) Structure of the RAP1 carboxy terminal (RCT) domain of RAP1 bound to the RAP1-binding motif (RBM) of TRF2 (PDB 3K6G) [32]. (**G**) Structure of the TRFH domain of TRF2 bound to a fragment of the BRCA1 carboxy terminal (BRCT) domain of RAP1 (residues 90–106) (PDB 4RQI) [68]. Dash lines represent disordered regions.

Interactions between POT1 and TPP1 have been characterised through crystal structures of TPP1 PBM in complex with the C-terminal region of POT1 encompassing the OB3 and HJRL domains (Figure 2A) [20,48]. Despite displaying an overall structural similarity to the archaeal Holliday junction resolvase Hjc, the HJRL domain of POT1 is incompatible with Holliday junction binding and does not possess any detectable Holliday junction resolvase activity [20,48,49]. The OB3 and HJRL domains of POT1 extensively interact with each other, forming an elongated architecture. TPP1 PBM adopts an extended helical conformation and binds across the OB3 and HJRL domains of POT1 (Figure 2A). The canonical ssDNA OB-fold binding groove of OB3 interacts with TPP1 PBM.

Cryo-electron microscopy (cryoEM) structures of the POT1-TPP1-TIN2 complex at 8–10 Å resolution have been reported [50]. The densities are fitted with crystal structures of the four domains of POT1 and TPP1 PBM (as described above), whereas TIN2 and the remainder of TPP1 are not resolved in the structure. POT1 domains adopt a Y-shaped conformation, with the OB3 and HJRL domains forming the base, and the OB1 and OB2 domains occupying the arms (Figure 2B). Movement of the OB1 and OB2 domains of POT1 relative to each other allows POT1 to adopt open and closed conformations (Figure 2B) [50]. The affinity of POT1 for DNA with a non-telomeric linker inserted between the OB1 (TTAGGG) and OB2 (TTAG) binding sites is similar to that for DNA where the two binding sites are adjacent [50]. Thus, the open and closed conformations may have a role in providing the flexibility in DNA binding.

### TIN2 — the structural scaffold

TIN2 consists of a telomere repeat factor homology (TRFH) domain, a TRFH-binding motif (TBM) and a mutations-in-dyskeratosis congenita (DC) domain (Figure 1D) [2]. Both TRF1 and TRF2 can bind the TBM of TIN2 [51]. TRF2 also binds the TRFH domain of TIN2, together with TPP1 [51,52]. A crystal structure of the TIN2 TRFH domain bound to TIN2-binding motif (TBM) of TPP1 and TRF2 reveals the molecular basis of these interactions (Figure 2C) [52]. Structurally, the TIN2 TRFH domain is similar to both the TRFH domains of TRF1 and TRF2 despite the low sequence identity (Figure 1D–F) [52,53]. TIN2 cooperatively interacts with TRF2 and TPP1 with the binding of one subunit increasing the affinity of TIN2 for the other subunit significantly [52]. This has possible implications in the assembly and stability of shelterin.

### TRF1 and TRF2 — the double-stranded binding proteins

Both TRF1 and TRF2 contain a TRFH domain that mediates homodimerisation and a Myb domain that binds to ds telomeric DNA (Figure 1E,F) [21,22]. The TRFH domains of TRF1 and TRF2 exhibit 27% sequence identity and are structurally similar (Figure 1E,F) [53]. However, differences in charge and the lengths of the interacting helices at the homodimerisation interface preclude heterodimerisation between TRF1 and TRF2. Nevertheless, the TRFH domains of TRF1 and TRF2 serve as platforms for protein-protein interactions [54–56]. For instance, the TRF1 TRFH domain interacts with the TBM of TIN2 (Figure 2D) [51]. Although the TIN2 TBM interacts with the TRFH domains of both TRF1 and TRF2 (Figure 2D,E), it has a 20-fold higher affinity for TRF1 than TRF2 [51]. This specificity is attributed to residues in TRF1 that are critical for TRF1-TIN2 interactions but absent in TRF2. Interestingly, the corresponding region in TRF2 interacts with the Apollo nuclease, which resects the newly replicated leading-strand telomeric DNA to generate the 3′ ss telomeric overhang [51,54,55,57–59]. Therefore, the difference in TRF1 and TRF2 dimerisation interface seems to confer specificity of interactions with shelterin accessory factors.

The Myb-domains of TRF1 and TRF2 exhibit remarkable similarities in their sequence, structure and DNA recognition [22,24]. The structures of the Myb domains of TRF1 and TRF2 show that both Myb domains bind the major DNA groove by recognising the TAGGGTT sequence motif (Figure 1E,F). Each structure also consists of a pair of the Myb domains, each binding on the opposite sides of the telomeric DNA (Figure 1E,F). Despite these similarities, recent studies reveal differences in their DNA recognition in a chromatin context as discussed below [45,46].

While the N-terminal domain of TRF1 is rich in acidic residues, the TRF2 counterpart is enriched in basic residues. The basic domain of TRF2 can bind four-way Holliday junctions, prevent Holliday junction resolvase recognition, and stabilise T-loops [60–63]. It also interacts with core histones, possibly enhancing the stability of telomeric nucleosomes [64]. In contrast, the acidic domain of TRF1 prevents TRF1 from inducing supercoiling and promoting strand invasion, properties typically associated with TRF2 [65–67]. Hence, the N-terminal domains of TRF1 and TRF2 play distinct roles in shaping the functions of TRF1 and TRF2, respectively, within cellular processes.

### RAP1 — TRF2 accessory factor

RAP1 comprises a BRCA1 carboxy terminal (BRCT) domain, a Myb domain, a coiled region and a RAP1 carboxy terminal (RCT) domain (Figure 1G). The RCT domain of RAP1 forms hydrophobic interactions with the RAP1-binding motif (RBM) of TRF2 (Figure 2F) [32]. A secondary interaction between the RAP1 BRCT and the TRF2 TRFH domain has been observed (Figure 2G) [68]. Notably, the motif within BRCT of RAP1 that is involved in interaction with TRF2 bears similarities to the TRF2-interacting peptide of Apollo nuclease [51]. The presence of this secondary interaction may alter the binding properties of the TRF2-RAP1 complex towards other interacting partners.

Besides RAP1, BRCT domains are prevalent in various proteins. They possess phosphor-peptide recognition ability and are often involved in DDR pathways [69]. The solution NMR structure of the human RAP1 BRCT domain revealed a distinctive architecture, featuring a layer of α-helices and a layer of β-sheets (Figure 1G) (PDB 7OZ0). This differs from the canonical BRCT domain, which typically exists as a sandwich of two α-helix layers with a β-sheet in between. Moreover, many BRCT domains occur in tandems, whereas each RAP1 only has one individual BRCT domain [69]. The Myb domain of RAP1 (Figure 1G), unlike that of TRF1 and TRF2, does not engage in DNA binding, attributed to its surface lacking positively charged residues [70].

### Reconstitution and architecture of large shelterin assemblies

Currently, structures of shelterin complex consisting of all six subunits or large shelterin subcomplexes remain elusive. Nonetheless, efforts have been made to reconstitute large shelterin assemblies [71–73]. Lim et al., 2017 reconstituted a shelterin complex lacking TRF1 and proposed a 1:1:1:2:2 stoichiometry of POT1:TPP1:TIN2:TRF2:RAP1. Recently, Zinder et al., 2022 characterised six-membered shelterin and subcomplexes using negative-stain electron microscopy, mass photometry and native mass spectrometry. Two-dimensional class averages of the complexes revealed significant conformational heterogeneity and flexibility [73]. The purified shelterin exists as not only a dimer containing two copies of each shelterin subunit but also various subcomplexes. This dimeric composition also aligns with the 2:2:2 stoichiometry of the TRF1-TIN2-TPP1 complex determined by native mass spectrometry [46]. The compositional and conformational heterogeneity of shelterin would necessitate further technological developments for structure determination of shelterin.

### Shelterin assembly

Current structural and biochemical data suggest that the assembly of shelterin subunits on telomeres occurs in a hierarchical manner [74,75]. This process likely begins with the binding of TRF1 and TRF2 to ds telomeric DNA [21–24]. TIN2 is then recruited to telomeres through its interactions with TRF1 and TRF2, further stabilising their association with the telomeric DNA. [38,39,76,77]. Subsequently, TIN2 facilitates the assembly of POT1 and TPP1 via direct interaction with TPP1 [13,40,76]. Although POT1 can bind to ss telomeric DNA and the ds-ss junction, it requires TPP1 for its recruitment to the telomeres [5,8,19,37,78]. RAP1 is recruited to telomeres through its association with TRF2 [35,36]. Interestingly, TRF1, TRF2, TIN2 and RAP1 are more abundant than POT1 and TPP1 in cells, suggesting that only a small fraction of shelterin complexes would contain all six subunits [79]. While it seems logical to assume that TRF1, TRF2, TIN2, and RAP1 form a core structural component of the shelterin complex, evidence indicates the existence of other shelterin subcomplexes [71–73]. The modular interactions between shelterin subunits support this compositional plasticity, allowing for variations in the assembly and function of the shelterin complex.

## The interplay between shelterin and the telomeric chromatin

### Telomeric nucleosomes and telomeric chromatin

**Figure 3.**
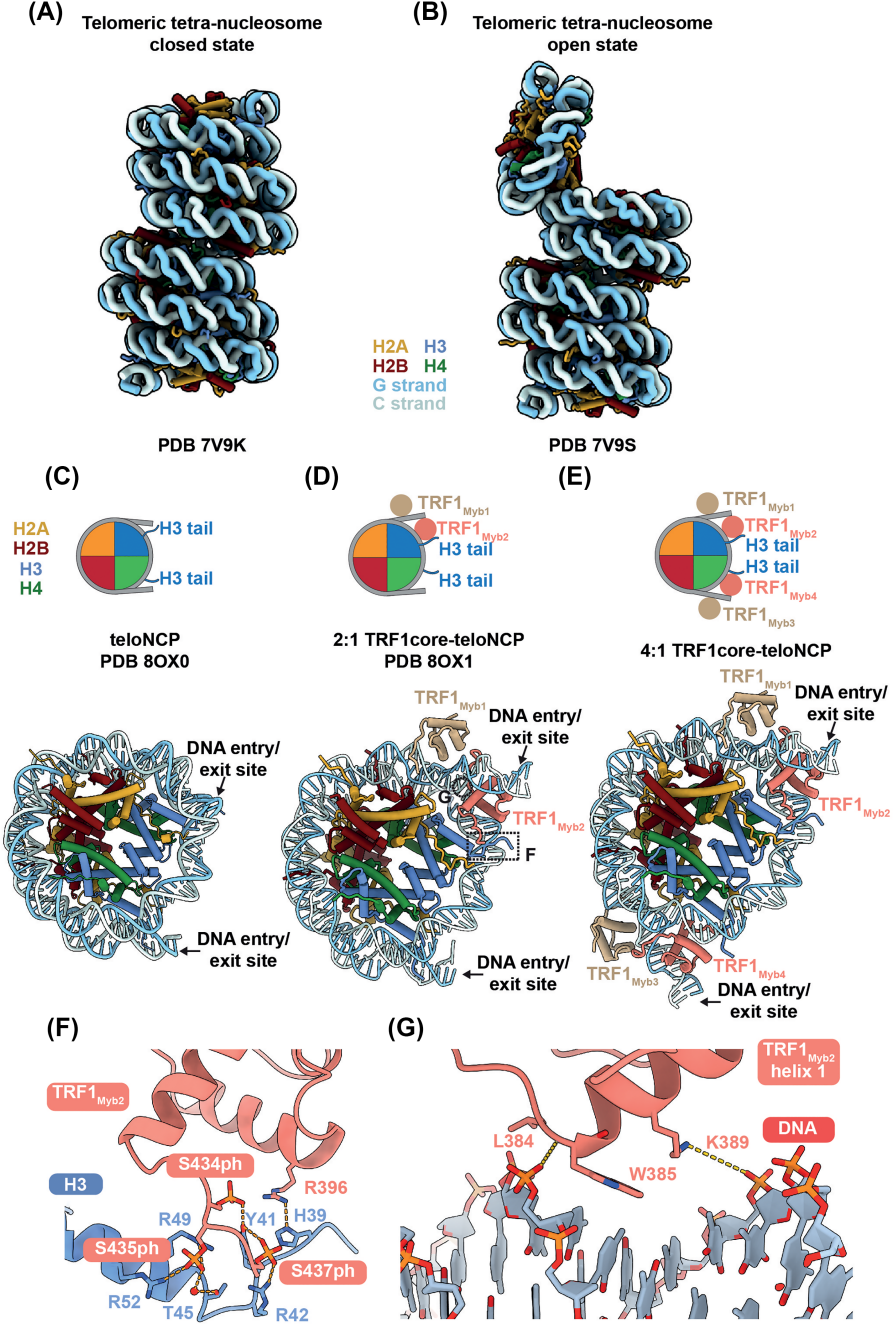
CryoEM structures of telomeric nucleosomes and chromatin. (**A**) Telomeric tetra-nucleosome shows columnar stacking of the chromatin (PDB 7V9K) [87]. (**B**) The ‘open state’ of telomeric tetra-nucleosome (PDB 7V9S) [87]. (**C**) Structure of a telomeric nucleosome (PDB 8OX0) [46]. (**D**) Structure of the 2:1 TRF1_core_-teloNCP complex with two copies of TRF1 Myb domain bound to the telomeric nucleosome (PDB 8OX1) [46]. Regions highlighted in panels F and G are indicated. (**E**) Structure of the 4:1 TRF1-teloNCP complex, with four copies of TRF1 Myb domain bound to the telomeric nucleosome (model fitted into EMD-17253) [46]. (**F**) Detailed view of the interactions between the C-terminal region of TRF1 Myb domain and histone H3 within the 2:1 TRF1_core_-teloNCP complex shown in panel (D). (**G**) Detailed view of the interactions between TRF1 Myb helix 1 and inner gyre DNA of the telomeric nucleosome within the 2:1 TRF1_core_-teloNCP complex shown in panel (D).

The hexameric TTAGGG repeat, characteristic of mammalian telomeres, is out of phase with the DNA helical turn [∼10 base-pairs (bp)], rendering it less conducive to nucleosome formation [80]. *In vitro* reconstitution of nucleosomes demonstrated that telomeric sequences exhibit higher free energy and lower affinity for binding to histone octamers compared with other DNA sequences tested [81,82]. *In vivo* studies using yeast mini-chromosome systems have similarly demonstrated that telomeric repeats disfavour nucleosome formation [83]. Despite these findings, the reported structures of telomeric nucleosome core particles (teloNCP) display a canonical nucleosome structure (Figure 3C) [46,84,85].

Furthermore, telomeric nucleosomes are intrinsically mobile, capable of sliding along telomeric DNA without the aid of chromatin remodellers [82,86]. Consistent with these findings, telomeric nucleosome arrays exhibit greater structural heterogeneity compared with arrays formed with the strong positioning Widom601 sequence [87].

The higher order structure of a telomeric chromatin differs from the bulk chromatin due to its relatively short nucleosome repeat lengths [43,44]. Two types of structural models have been proposed for telomeric chromatin: one with a zig-zag type nucleosome arrangement [88], and the other with a column-like model formed via nucleosome stacking [89]. A recent cryoEM study supports the latter model by demonstrating that *in vitro* reconstituted telomeric chromatin forms a tightly packed columnar structure (Figure 3A) [87]. Columnar stacking of telomeric nucleosomes is mediated by multiple histone tails, including the C-terminal tail of H2A, the N-terminal helix of H3 and the N-terminal tail of H4. This columnar structure represents a ‘closed state’ of telomeric chromatin. An ‘open state’ of telomeric chromatin was also identified, where one nucleosome is nearly vertical to the rest of the chromatin stack (Figure 3B). This ‘open state’ exposes the acidic patch of the nucleosome, which serves as a common interaction platform for numerous chromatin binding proteins [90]. Therefore, the switch from the ‘closed’ to ‘open state’ of telomeric chromatin may enable telomeric chromatin to interact with shelterin and other complexes involved in telomere regulation.

In the cryoEM structure of the columnar telomeric chromatin, telomeric chromatin adopts a short nucleosome repeat length of ∼132 ± 2 bp [87]. Although it is consistent with previous studies which suggest that telomeric chromatin is more compact than bulk chromatin, nucleosome repeat lengths of mammalian telomeric chromatin estimated from digestion experiments range from 150 to 165 bp [43,44,91]. Telomeric DNA-associated proteins, such as shelterin, likely contribute to the disparity in the nucleosome repeat lengths between the *in vitro* reconstituted telomeric chromatin and telomeric chromatin isolated from cells.

In telomeric chromatin, histone H1 is present but less abundant compared with bulk chromatin [91,92]. The compact columnar state of telomeric chromatin observed in the cryoEM structure is incompatible with the presence of histone H1, which may partially explain its underrepresentation in telomeric chromatin [46,87]. A proteomic study suggests that histone H1 is enriched only during telomere replication and may contribute to telomere stability [93].

### Interactions between shelterin complex and telomeric chromatin

#### TRF1 interaction with the telomeric chromatin

As discussed earlier, TRF1 and TRF2 bind ds telomeric repeats in a sequence-specific manner. Although most previous studies utilised linear dsDNA, a few examined the interactions between the two TRF proteins and telomeric nucleosomes [45,46,94–97]. TRF1 can bind telomeric repeats in a nucleosomal context, showing a preference for solvent-exposed binding sites [94]. TRF1 exhibits lower binding affinity towards a trypsinised nucleosome (with all histone tails removed), indicating a role for the histone tails in mediating the interactions between TRF1 and telomeric nucleosomes [45]. TRF1 binding also induces mobility of telomeric nucleosomes, potentially altering the spacing between them [45,94,95].

The structures of a teloNCP in complex with a shelterin subcomplex consisting of TRF1, TIN2 and TPP1 (named TRF1_core_) were determined using cryoEM [46]. In one structure, a pair of the Myb domains of TRF1 binds to one entry/exit site of the nucleosome, whilst the other structure contains two pairs of the Myb domains of TRF1 binding to both entry/exit sites (Figure 3D,E). Binding of TRF1 results in the unwrapping of the entry/exit sites of the teloNCP as well as a 1-bp shift in the register of the DNA positioning on the nucleosome compared with the apo-teloNCP structure (Figure 3C,D). The observed ability of TRF1 to modulate nucleosomes is consistent with previous data showing that TRF1 is capable of sliding telomeric nucleosomes [95].

The sequence-specific interactions between TRF1 and telomeric DNA mostly resemble those observed in the previous crystal structure of TRF1 Myb domain bound to linear telomeric DNA (Figure 1E) [24]. However, the cryoEM structures reveal two distinct interaction interfaces specific to TRF1-nucleosome contacts [46]. First, the C-terminal region of TRF1 Myb domain interacts with the histone H3 tail, with three phosphorylated serine residues of TRF1 mediating these interactions (Figure 3F). Mutating these serine residues to alanine abrogates the binding of TRF1 to the teloNCP. Second, helix 1 of TRF1 Myb domain, which does not participate in the interactions between TRF1 and linear telomeric DNA, binds to the inner gyre of nucleosomal DNA (Figure 3G). Collectively, the TRF1_core_-teloNCP structures highlight the previously overlooked role of nucleosomes in mediating shelterin interactions with telomeres.

#### TRF2 interaction with the telomeric chromatin

In contrast with TRF1, the binding of TRF2 to telomeric repeats is strongly hampered in the context of a nucleosome [45]. This disparity is attributed to the distinct N-terminal domains of TRF1 and TRF2. Deletion and domain swapping experiments revealed that deleting the N-terminal basic domain of TRF2 or substituting it with the acidic domain of TRF1 enhances its binding to nucleosomes [45]. Key nucleosome-interacting residues in TRF1 Myb domain, identified within TRF1_core_-teloNCP structure, (Figure 3F,G) are not conserved in TRF2. This discrepancy further contributes to the different binding properties of TRF1 and TRF2 towards telomeric nucleosomes [46]. Another study suggests interactions between the basic N-terminal domain of TRF2 and core histone proteins [64]. However, in this study, the histone proteins were not assembled into nucleosomes, which may explain the discrepancy in TRF2 behaviour compared with findings of Galati et al., 2015.

Despite its relatively weak binding to telomeric nucleosomes, overexpression of TRF2 reduces nucleosome density and alters nucleosome spacing in the telomeric region *in vivo* and *in vitro* [96]. In contrast with these findings, other studies demonstrate that TRF2 binds to *in vitro* reconstituted telomeric nucleosome arrays and induce the chromatin compaction [97–99]. Hence, further studies are necessary to reconcile these contradictory results.

#### Organisation of shelterin complex and nucleosomes on telomeric chromatin

Despite the lack of structures of either entire shelterin complex or shelterin in complex with telomeric nucleosomes/chromatin, we can speculate on the interactions between shelterin and telomeric nucleosomes based on current data. The unwrapping of the nucleosome entry/exit sites by TRF1 as observed in the TRF1_core_-teloNCP structure (Figure 3D,E) resembles that by pioneer transcription factors, chromatin remodellers and histone-modifying enzymes [100–102]. Such unwrapping facilitates the opening of the local chromatin environment, allowing the access of protein complexes to this chromatin region. Similarly, TRF1 may induce the ‘open state’ of telomeric chromatin and potentially allow access of other shelterin components or protein complexes to telomeric chromatin during processes such as replication and transcription.

**Figure 4.**
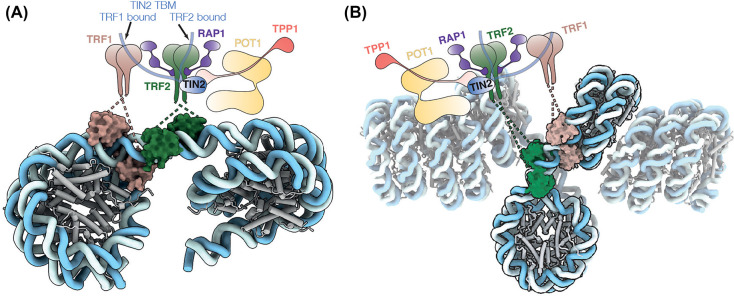
Proposed structural remodelling of the telomeric chromatin by shelterin. (**A**) Proposed model for shelterin-telomeric nucleosome interactions. In this model, there is a linker between the nucleosomes based on the nucleosome repeat length estimated *in vivo* [43,44]. TRF1 would bind the nucleosome-linker junction; while TRF2 would occupy the linker region. (**B**) Proposed model for shelterin-chromatin interactions. In this model, shelterin would bind the open region of telomeric chromatin in a similar manner as shown in panel (**A**).

Shelterin potentially binds the junction between a flipped-out nucleosome and the columnar nucleosome stack (Figure 4B). In this configuration, TRF1 would occupy the nucleosome entry/exit site, whereas the hindrance of binding of TRF2 to a nucleosome indicates that TRF2 preferentially binds the linker DNA (Figure 4). This binding mode would be compatible with the ‘open state’ of telomeric chromatin and could account for the discrepancy in the nucleosome repeat length of the *in vitro* reconstituted telomeric chromatin and that determined *in vivo* (Figure 4B) [43,44,87]. Whether other shelterin components, aside from TRF1 and TRF2, may also contribute to nucleosome interactions remains unknown. One possibility is that the acidic patch of the flipped-out nucleosome in the telomeric chromatin might be neutralised by shelterin components, thus preventing the reformation of the nucleosome stacking.

### Regulations of shelterin-chromatin interactions

#### Cell cycle regulations of shelterin-chromatin interactions

The ‘open state’ of telomeric chromatin observed by Soman et al., 2022 suggests the dynamic nature of telomeric chromatin akin to other chromatin regions (Figure 3B). The interplay between shelterin and chromatin likely plays a crucial role in regulating telomeric chromatin dynamics, further influenced by various factors such as cell-cycle regulation.

Our structural investigation of the TRF1_core_-teloNCP complex reveals the essential role of phosphorylation at S434, S435 and S437 on the C-terminus of TRF1 for nucleosome binding (Figure 3F) [46]. Another study identified the Polo-like kinase 1 (Plk1), a key regulator of the cell-cycle, being responsible for phosphorylation of S435 of TRF1 [103]. This phosphorylation event modulates TRF1 association with telomeres, which varies across different cell-cycle stages and peaks during mitosis [103]. Therefore, the phosphorylation state of TRF1 C-terminal region may affect the interactions between TRF1 and telomeric chromatin in a cell-cycle specific manner.

Additionally, interactions between TRF2 and telomeric chromatin are thought to be cell-cycle regulated. Overexpression of TRF2 does not affect telomeric nucleosome occupancy at the end of G1 phase but mediates the reduction of occupancy in subsequent cell-cycle phases [96]. This implies a potential role of TRF2 in shaping chromatin architecture during and after telomere replication, potentially contributing to telomeric chromatin organisation.

It is plausible that shelterin or at least some of its components associates or dissociates from telomeric chromatin during different cell-cycle stages. Shelterin thereby regulates telomere function by contributing to the organisation and dynamics of telomeric chromatin in a time-dependent manner. It will be interesting to study the composition of shelterin bound to telomeric chromatin at different stages of cell cycle to provide a deeper insight into the regulation of shelterin-chromatin interactions during the cell cycle.

#### Epigenetic regulation of shelterin-chromatin interactions

Shelterin-chromatin interactions are likely subject to various epigenetic regulations, such as histone modifications and deposition of histone variants. Telomeric chromatin is traditionally viewed as heterochromatic [104]. However, in human telomeric chromatin, the heterochromatic H3K9me3 marks are not enriched, while both active and repressive histone marks coexist [105]. Various histone modifications and histone variants are present in telomeres, such as H4K20 methylations, H3K56 acetylation, histone H3.3 and histone H2A.X variants [105–108].

Histone modifications or variable regions across histone variants that are positioned in proximity to the shelterin-chromatin interfaces could potentially affect their interactions. In the TRF1_core_-teloNCP structure, TRF1 Myb is in close proximity to the N-terminal helix (residues 45–56) of histone H3 and the C-terminal tail of H2A [46]. Histone modifications in this region, such as H3T45 phosphorylation, H3K56 acetylation, and ubiquitination at H2AK119 and H2AK120, could impact TRF1 nucleosome binding [46]. In the cryoEM structures of telomeric chromatin, various histone tails mediate nucleosomal stacking in the columnar structure [87]. Histone modifications, including acetylations at H3K56, H4K12, H4K16 sites and methylations at H3K9, H3K79, H4K20 sites, and histone variant H2AX would potentially reshape the columnar architecture of telomeric chromatin [87]. Therefore, epigenetic regulation of telomeric chromatin could influence the accessibility of telomeric complexes, such as shelterin.

## Outlook

In recent years, the Resolution Revolution in cryoEM has enabled the determination of complex macromolecular structures such as telomerase, another key player in telomere maintenance [109,110]. However, uncovering structures of shelterin or its large subcomplexes remains a formidable task. Several challenges contribute to this obstacle. Firstly, interactions between different shelterin domains primarily rely on domain-peptide interactions (Figure 2), with affinities of several shelterin subunit interactions in the micromolar range [51,52,75]. Secondly, shelterin subunits feature numerous unstructured linker regions between their folded domains (Figure 1B–G), likely contributing to the conformational heterogeneity of shelterin [46,50,73]. In fact, the dynamic nature of shelterin has been suggested to be physiological, enabling it to fulfil diverse roles at telomeres [73]. Thirdly, variability arises from protein isoforms and post-translational modifications of shelterin proteins [42,46,111–115]. Additionally, as discussed above, *in vitro* reconstitution and *in vivo* studies suggest variability in subunit stoichiometries, indicating that shelterin may mostly exist as subcomplexes [71,73,79]. The subunit composition and conformation of shelterin are likely influenced by the telomere landscape, including factors such as interaction partners, linear ds telomeric DNA, the ds-ssDNA junction, the ssDNA overhang, and T-loops or telomeric chromatin. Therefore, technological advancements in cryoEM sample preparation and heterogeneity analyses, combined with consideration of context-specific telomeric substrates for shelterin will be crucial for achieving a comprehensive understanding of shelterin structure and dynamics.

## Perspectives

- Shelterin serves a critical role in safeguarding the ends of mammalian chromosomes, preventing them from being identified as DNA break sites and thereby preserving genome stability.
- Numerous structural studies unveiled structures of various domains within shelterin subunits and elucidated the molecular basis of several inter-subunit interactions within shelterin, as well as interactions with telomeric nucleosomes. However, the complete structures of the shelterin complex or large shelterin subcomplexes remain elusive.
- Overcoming the conformational and compositional heterogeneity of the shelterin complex is crucial for achieving a comprehensive molecular understanding of its structure and function.

## Open Access

Open access for this article was enabled by the participation of University of Cambridge in an all-inclusive *Read & Publish* agreement with Portland Press and the Biochemical Society under a transformative agreement with JISC.

## Abbreviations

BRCT: BRCA1 carboxy terminal
cryoEM: cryo-electron microscopy
DDR: DNA damage response
ds: double-stranded
HJRL: Holliday junction resolvase-like
OB: oligosaccharide/oligonucleotide binding
PBM: POT1-binding motif
RBM: RAP1-binding motif
RCT: RAP1 carboxy terminal
ss: single-stranded
TBM: TIN2-binding motif or TRFH-binding motif
teloNCP: telomeric nucleosome core particle
TRFH: telomeric repeat factor homology
